# Diosgenin and Its Fenugreek Based Biological Matrix Affect Insulin Resistance and Anabolic Hormones in a Rat Based Insulin Resistance Model

**DOI:** 10.1155/2019/7213913

**Published:** 2019-04-04

**Authors:** Rita Kiss, Georgina Pesti-Asbóth, Mária Magdolna Szarvas, László Stündl, Zoltán Cziáky, Csaba Hegedűs, Diána Kovács, Andrea Badale, Endre Máthé, Zoltán Szilvássy, Judit Remenyik

**Affiliations:** ^1^Department of Pharmacology and Pharmacotherapy, Faculty of Medicine, University of Debrecen, Nagyerdei krt. 98, 4032 Debrecen, Hungary; ^2^Institute of Food Technology, Faculty of Agricultural and Food Sciences and Environmental Management, University of Debrecen, Böszörményi út 138, 4032 Debrecen, Hungary; ^3^Agricultural and Molecular Research and Service Institute, University of Nyíregyháza, Sóstói út 31/B, 4400 Nyíregyháza, Hungary; ^4^Cera-Med Ltd., Kútvölgyi u. 1, 4225 Debrecen, Hungary

## Abstract

Fenugreek is known since ancient times as a traditional herbal medicine of its multiple beneficial effects. Fenugreek's most studied and employed effect is its hypoglycemic property, but it can also be useful for the treatment of certain thyroid disorders or for the treatment of anorexia. The regulation of glucose homeostasis is a complex mechanism, dependent on the interaction of different types of hormones and neurotransmitters or other compounds. For the study of how diosgenin and fenugreek seeds modify insulin sensitivity, we used a rat insulin resistance model induced by high-fat diet. Diosgenin in three different doses (1mg/bwkg, 10mg/bwkg, and 50 mg/bwkg, respectively) and fenugreek seed (0.2 g/bwkg) were administered orally for 6 weeks. Insulin sensitivity was determined by hyperinsulinemic euglycemic glucose clamp method. Our research group found that although glucose infusion rate was not significantly modified in either group, the increased insulin sensitivity index and high metabolic clearance rate of insulin found in the 1 mg/kg diosgenin and the fenugreek seed treated group suggested an improved peripheral insulin sensitivity. Results from the 10 mg/kg diosgenin group, however, suggest a marked insulin resistance. Fenugreek seed therapy results on the investigated anabolic hormones support the theory that, besides insulin and gastrointestinal peptides, the hypothalamic-hypopituitary axis regulated hormones synchronized action with IGF-1 also play an important role in the maintaining of normal glucose levels. Both diosgenin and fenugreek seeds are capable of interacting with substrates of the above-mentioned regulatory mechanisms, inducing serious hormonal disorders. Moreover, fenugreek seeds showed the ability to reduce the thyroid hormone levels at the periphery and to modify the T4/T3 ratio. It means that in healthy people this effect could be considered a severe side effect; however, in hypothyroidism this effect represents a possibility of alternative natural therapy.

## 1. Introduction

Fenugreek (*Trigonella foenum-graecum*) appears to be rich in phytonutrients with multiple pharmacological effects [1–4]. Extracts have been made using fenugreek vegetative organs and/or seeds, and multiple* in vivo* and* in vitro* studies revealed analgesic [5, 6], hepatoprotective [7, 8], and hypolipidemic [9–14] effects. It was suggested that the hypolipidemic effects could be put on the expense of saponins that transformed into sapogenins in the gastrointestinal tract would trigger the reduction of serum cholesterol levels [15, 16]. Among saponins, the diosgenin was found to induce changes in the lipid profile of different tissues or organs, such as plasma, liver, heart, or brain in a diabetic rat model [17], indicating that these modifications might be correlated with a hypoglycemic effect. In another study, it was demonstrated that both fenugreek and diosgenin treatment prevented high-fat, high-sugar diet-induced endothelial dysfunction, and redox changes [18]. Besides diosgenin, the 4-hydroxyisoleucine (4-HIL), a fairly rare amino acid found in fenugreek, was shown to increase insulin secretion upon glucose stimulation [19, 20]. Moreover, 4-HIL could reduce insulin resistance (IR) in muscle and/or liver by stimulating phosphoinositide 3 (PI3) kinase activity [21]. Interestingly, increased insulin receptor [22], adiponectin, and PPAR*γ* [23] expression were also associated with the IR reducing effects of fenugreek. These results, together with the fenugreek seed administration induced improvement of hepatic steatosis, strongly plead for a cause-and-effect type of relationship between liver specific IR and steatosis [23]. It is also demonstrated that the health promoting effect of fenugreek is mostly due to its antioxidant capacity [24]. Among phytonutrients, the polyphenols from fenugreek seeds were shown to exert antioxidant properties by inhibiting lipid peroxidation [8]. A fenugreek seed and/or seed extracts were found to increase glucose uptake; reduced glycosylated haemoglobin levels together with proinflammatory cytokines and pancreatic enzymes; in dose-dependent manner restored the glycogen levels in muscle and liver; inhibited lipid peroxidation; and reinstated some antioxidant enzymes-glutathione (GSH) and superoxide dismutase (SOD) activities in the liver and pancreas [25]. It has also been suggested that, in the case of fenugreek, the antioxidant potential complements the hypolipidemic, hypoglycemic, and anti-inflammatory effects [26]. Accordingly, diabetic rats treated with fenugreek showed reduced serum glucose levels, and the activities of antioxidant defence enzymes increased due to the expression of the mentioned genes in the liver or/and brain [27]. Another study concluded that fenugreek seed extract could efficiently suppress testicular oxidative stress in combination with apoptosis and inflammation [28]. Interestingly, a comparative study showed substantial differences between the antioxidant activity of fenugreek leaf and seed extracts with respect to the DNA damage protective activities [29]. Another study was indicating a functional link between the antioxidant and anticancer effects of fenugreek [30]. Nevertheless, the anticancer effect of fenugreek has been thoroughly assessed [31–33], and selective cytotoxicity has been observed in case of various cancer cell lines. Thus, for example, in the case of breast cancer, T cell lymphoma, B cell lymphoma, and thyroid papillary carcinoma cell lines, significant cytotoxic effects were observed while no significant cytotoxicity was evident for normal human cells. [28]. Furthermore, Vígh et al. were able to correlate the chemical composition of fenugreek seed extracts with reduced viability of the T-47D and ZR-75-1 breast cancer cell lines [34]. Moreover, they could also show that an aqueous fenugreek seed extract could affect the viability of cancerous cells in a dose-dependent antagonistic fashion. However, only diosgenin from fenugreek was proven to efficiently reduce cancer cell viability, while inducing apoptosis [35]. More specifically, it was ascertained that diosgenin inhibited telomerase activity by downregulating the hTERT gene expression could reverse multidrug resistance in cancer cells by sensitizing them to standard chemotherapy, activating p53 gene and the STAT3 signalling pathway [36–38]. In addition to fenugreek beneficial properties, some toxic effects were also reported related to embryonic development, spermatogenesis, allergic reactions, neurotoxicity, and altered levels of thyroid hormones or inhibition of hematopoietic regulatory elements [39].

Taken together, plethora of research has been conducted to analyze independently the fenugreek composition and the generated biological effects, yet studies to relate composition to function are available in limited numbers, mostly due to the limitation of research models and methodology. Among the constituents of fenugreek, diosgenin stands at the forefront of current research, and results are indicating multiple biological effects depending on the characteristics of assessed models. Our research reported in current paper describes a comparative dose-dependent study based on fenugreek and diosgenin in conjunction with an improved peripheral insulin sensitivity and the interference with hypothalamic-hypopituitary axis regulated hormones. To the best of our knowledge there is no published evidence suggesting the interdependence between the possible concentration-dependent toxic effects of fenugreek treatment and the most important anabolic hormones, such as the growth hormone, IGF-1, T3, and T4. Our results further demonstrate the importance of careful dose-dependent assessment of fenugreek specific pharmacological effects. 

## 2. Materials and Methods

### 2.1. Analytical Examination of Fenugreek Seeds 

#### 2.1.1. Preparation of Extract

The seeds of fenugreek were obtained from an authorized local distributor. Powdered fenugreek seeds were extracted with water: ethanol mixture (1:1) for 72 h at 70°C using Soxhlet apparatus. This extract was then concentrated to dryness by removing the solvent in the rotary evaporator under reduced pressure.

#### 2.1.2. Preparation of Sample for Analysis

One gram of thoroughly milled fenugreek seeds was accurately weighed. To the fine powder obtained, 80 mL of 3 M hydrochloric acid was added, and then it was kept on reflux for 1 h on water bath at 100°C. Mixture was allowed to cool at room temperature and was diluted further up to the mark with water. Diethyl ether was used for the extraction process of the mixture. After that the ether layer was separated and left to evaporate. The remained residue was dissolved in 25 mL of methanol. This resulting solution was used as test solution [40].

#### 2.1.3. Chromatographic Conditions for Ultra High Performance Liquid Chromatography

**Figure 1:**
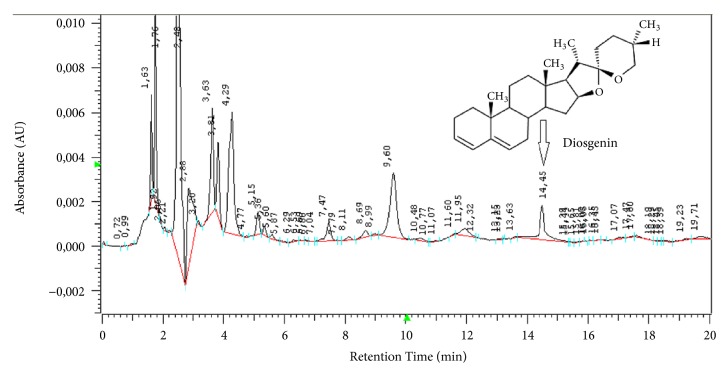
Chromatogram of fenugreek (*Trigonella foenum-graecum L*.).

**Figure 2:**
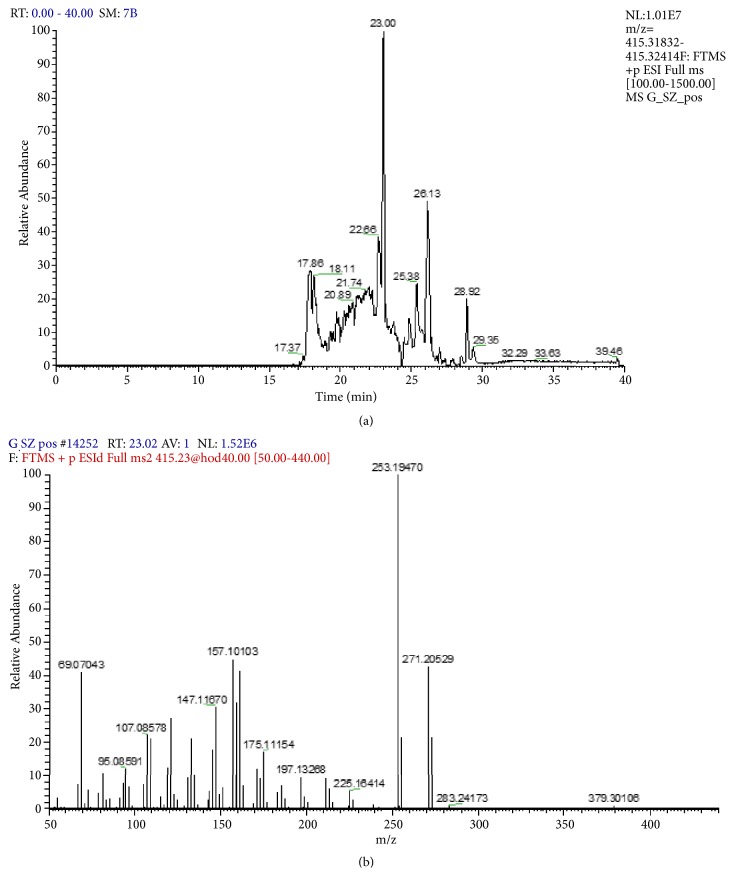
Total ion chromatogram of alcoholic extract of fenugreek (*Trigonella Foenum-graecum L*.) in negative ionization mode (a) including fragmentation spectra of the parent ion (b).

The preparation of diosgenin was done with Chromaster Rs ultra high performance liquid chromatography system (with a 6430 diode array detector (DA), 6270 autosampler, 6170 binary pump, 6310 column oven, software Agilent Open LAB chromatography data system). Diosgenin was separated on a reverse-phase 10 mm × 4.6 mm x 2.5 *μ*m Kinetex XB-C18 column. The mobile phase was prepared from water (solvent A) and acetonitrile (solvent B). The mobile phase was degassed and filtered through 0.45 *μ*m filter before use. The gradient program used was from A to B (10:90 v/v); 20-21 min, linear change from A-B (10:90 v/v) to A-B (2:98 v/v); 21-25 min, constant change from A-B (2:98 v/v); 25-26 min, linear changes from A-B (2:98 v/v) to A-B (10-90 v/v); and 26-30 min, constant change from A to B (10-90 v/v). The flow rate of the mobile phase was 0.7 mL/min. The temperature of the column was set to 25°C. The injection volume was 10 *μ*L. The DA was set at 254 nm to acquire the chromatogram. Diosgenin was identified by comparing the retention time and spectra obtained from sample and standard solutions (see Figure 1) [1]. We performed a total ion chromatogram of fenugreek (alcoholic extract) in negative ionization mode including fragmentation spectra of the parent ion (see Figure 2) [40, 41].

### 2.2. Animals and Protocols

#### 2.2.1. Ethics

The study was approved by and was in accordance with the guidelines of and the Animal Ethics Committee of the University of Debrecen (25/2013 DE MÁB).

#### 2.2.2. Animals

Male Wistar rats (n=42) were used throughout the study. Rats were maintained in a controlled environment (22-24°C, 12-12 h light/dark cycle). For the animals a week of acclimation period was provided; thereafter the rats were randomly selected into six experimental groups: healthy control (C), high-fat diet control (HF), high-fat diet + 1 mg/kg diosgenin (1D), high-fat diet + 10 mg/kg diosgenin (10D), high-fat diet + 50 mg/kg diosgenin (50D), and high-fat diet + 0,2 mg/kg fenugreek seed (FG). The healthy control rats had access to a standard laboratory chow (S8106-S011 SM R/M-Z+H, ssniff Spezialdiäten GmbH, Germany) and fresh tap water ad libitum. Animals in the five other groups received ad libitum a diet with high proportion of crude fats and carbohydrates, defined as high-fat diet (HFD) and 5% sucrose in the drinking water. This special rodent chow (“824018-45%AFE FAT”) was provided by “Special Diets Services”, UK. For the treated groups, the previously mentioned diosgenin doses, or the thoroughly milled fenugreek seeds were incorporated into the food. As the endpoint of the experiment the seventh week of treatment period was determined.

#### 2.2.3. Hyperinsulinemic Euglycemic Glucose Clamp (HEGC)

The hyperinsulinemic euglycemic glucose clamp (HEGC) method is a well-known and the most acceptance investigation to determine the exact and precise insulin sensitivity of insulin dependent tissues described by DeFronzo et al. [42, 43]. According to our previously configured and validated method to measure whole body insulin sensitivity [44] we performed the HEGC method on each animal of our current investigation. The animals needed to be anaesthetized before the procedure. After an overnight starvation, general anaesthesia was induced and sustained by an intraperitoneal injection of 50 mg/kg sodium thiopental (Thiopental Sandoz®, Sandoz Pharmaceutical PLC, Switzerland). To allow the spontaneous breathing of the animals, a polyethylene tube was inserted into the trachea. The HEGC method presumes two separate venous infusion lines for the administration of the insulin and glucose solution and an arterial cannula that serves for blood sampling for the subsequent determination of blood glucose levels and for monitoring the blood pressure during the whole period of the investigation. Two branches of the jugular vein and same side carotid artery were exposed and cannulated. After thirty-minute stabilization period continuous insulin (Humulin R®, Eli Lilly, Indianapolis, IN, USA) and 20% w/v glucose infusion were initiated simultaneously through the jugular vein. The rate of insulin infusion was set to 3 mU/kg/min while the glucose infusion rate was adjusted in accordance with maintaining the euglycemic (5.5 ± 0.5 mmol/l) blood glucose status. The blood glucose concentration was determined before the starting of the infusions (insulin and glucose respectively), during the first 80 minutes of the experiment in 5-minute intervals, and at every 10 minutes after the “steady state” condition developed, using a blood glucose testing device (Accu-Chek, Roche Diagnostics, Budaörs, Hungary). In addition, the fasting and steady state levels of insulin were determined from the plasma obtained from blood samples collected in EDTA tubes (0.5 ml, in 20 *μ*l EDTA, 10 *μ*l Trasylol; Bayer, Leverkusen, Germany) before the initiation of insulin infusion and during the steady state period of the experiment. The blood samples were centrifuged for two minutes, at 10.000 g and 4°C (Centrifuge 5415R, Eppendorf GmbH, Germany), and the plasma was frozen and stored at -70°C for further determinations.

### 2.3. Sample Preparation and Determination of Hormone Levels

#### 2.3.1. Description of the Blood Sample Preparation

To determine the plasma concentration of insulin, IGF-1, T3, T4, and GH from each animal blood samples were collected into EDTA-coated blood collection tubes (BD, Franklin Lakes, NJ, USA). The samples were centrifuged at 3000 rpm for 10 min (Centrifuge 5418, nonrefrigerated, with rotor FA-45-18-11, 230V/50-60Hz, 36 ml, 5418 000.017). The plasma was then aliquoted and stored frozen at -20°C until analysis.

#### 2.3.2. Determination of Insulin

The concentration of insulin was determined using a commercially available ELISA kit (Insulin ELISA, Immuno Diagnostics, Woodland Hills, California, USA, kit number: 1606-15). The absorbance was measured at 450 nm colorimetrically. The insulin concentrations were determined by comparing the absorbance values of the samples to the standard curve. The insulin concentrations were expressed as plasma insulin concentration (in *μ*IU/mL). The reagents were prepared according to the manufacturer's instructions. 50 *μ*L samples were added to sample wells. The standard curve was prepared as it was described in the protocol and 50 *μ*L of the diluted standard solution was added to the appropriate wells. 100 *μ*L of the Enzyme Reagent was added to each well; then the microplate was swirled gently for 20-30 seconds to mix and covered with a plastic wrap and was incubated for 120 minutes at room temperature (20-27°C). The liquid of the microplate was discarded by decantation and tap and blot the plate dry with absorbent paper. 350 *μ*L of wash buffer was added, decanted, and aspirated, and this step was repeated two additional times for a total of three washes. 100 *μ*L of working substrate was added to solution in all wells and the microplate was incubated at room temperature for 15 minutes. Then 50 *μ*L of stop solution was added to each well and mixed gently 15-25 seconds. Absorbance was measured at 450 nm. Sample insulin concentration was compared with the insulin standard curve and the concentration of insulin was expressed as plasma insulin concentration (in *μ*IU/ml).

#### 2.3.3. Determination of Insulin-Like Growth Factor-1 (IGF-1)

The insulin-like growth factor-1 was determined using a commercially available ELISA kit (Eagle Biosciences, INC., Nashua, New Hampshire, kit number: IGF31-K01). The reagents were prepared according to the manufacturer's instructions. Standards and samples were prepared as described in the protocol book. 20 *μ*L of the samples and standard were added to the appropriate wells and 100 *μ*L of the biotinylated IGF was added to each well and the plate was incubated at room temperature for two hours. The solution was aspirated from the microplate and each well was rinsed with 300*μ*L of 1x Wash solution and repeated 3 times. 150 *μ*L of the enzyme complex was added to each well after the wash process and the plate was incubated one hour at room temperature. Then the wash process was repeated three times. 100 *μ*L of substrate was added to each well and incubated for 15 minutes. 100 *μ*L of stopping solution was added to each well. Absorbance was measured at 450 nm. Sample IGF-1 concentration was compared with the IGF-1 standard curve and the concentration of IGF-1 was expressed as plasma IGF-1 concentration (in ng/mL).

#### 2.3.4. Determination of Growth Hormone (GH)

The concentration of growth hormone was assessed with a commercially available assay kit (Elisa Cloud Immunoassay, Huston, USA, kit number: SEA044). In the first step, the wells were determined wells for diluted standards, blank, and samples. 100 *μ*L of each of dilutions standards, blank, and samples was added to the correct wells, then the plate was covered and incubated for 1 hour at 37°C. After incubation the liquid was removed from each well and 100 *μ*L of Detection Reagent A working solution was added to each well and incubated for 1 hour at 37°C. The plate was washed with 350 *μ*L of 1x wash solution and repeated 3 times. After the last wash any remaining wash buffer was removed by aspirating and 100 *μ*L of Detection Reagent B working solution was added to each well and incubated for 30 minutes at 37°C. The aspiration and wash process were repeated 5 times. After the washing process 90 *μ*L of Substrate Solution was added to each well; then the plate was covered and incubated for 10-20 minutes at 37°C. After incubation, 50 *μ*L of Stop Solution were added to each well. Absorbance was measured at 450 nm. Sample GH concentration was compared with the GH standard curve and the concentration of GH was expressed as plasma GH concentration (in pg/mL).

#### 2.3.5. Determination of Triiodothyronine (T3)

The concentration triiodothyronine was determined using a commercially available ELISA kit (Wuxi Donglin Sci & Tech Development Co., Ltd. Wuxi, Jiangsu, China, kit number: DL-T3-Ge). The color change was measured spectrophotometrically at a wavelength of 450 nm. The concentrations of T3 in the samples were determined by comparing the absorbance of the samples to the standard curve. The T3 concentrations of the samples were expressed as plasma T3 concentration (in ng/mL). The reagents were prepared according to the manufacturer's instructions. A standard curve was prepared; the diluted standard solutions, the blank, and the samples were added to the appropriate wells (50 *μ*l). 50 *μ*L of Detection A was also added to each well immediately and the plate was shaking gently and covered with a plate sealer and incubated for 1 hour at 37°C. The solution was aspirated from the microplate and each well was washed with 350*μ*L of 1x Wash solution 3 times. To each well then 100 *μ*L of Detection Reagent B was added; then the plate was incubated at 37°C for 1 hour. After the incubation, the aspiration/wash cycle was repeated 5 times; then 90 *μ*L of Substrate Solution was added to each well after the end of the wash process. The plate was incubated for 15-25°C minutes at 37°C; then 50 *μ*L of Stop solution was added to each well. Absorbance was measured at 450 nm. Sample T3 concentration was compared with the T3 standard curve and the concentration of T3 was expressed as plasma T3 concentration (in ng/mL).

#### 2.3.6. Determination of Thyroxine (T4)

The concentrations of thyroxine were determined using a commercially available ELISA kit (Wuxi Donglin Sci & Tech Development Co., Ltd. Wuxi, Jiangsu, China, kit number: DL-T4-Ge). The reagents were prepared according to the manufacturer's instructions. A standard curve was prepared; the diluted standard solutions, the blank, and the samples were added to the appropriate wells (50 *μ*l). 50 *μ*L of Detection A was also added to each well immediately and the plate was shaking gently and covered with a plate sealer and incubated at 37°C for 1 hour. The solution was aspirated from microplate. Each well was washed with 350*μ*L of 1x Wash solution 3 times. To each well then 100 *μ*L of Detection Reagent B was added and at 37°C the plate was incubated for 1 hour. After the incubation, the aspiration/wash cycle was repeated 5 times; then 90 *μ*L of Substrate Solution was added to each well after the end of the wash process. The plate was incubated for 15-25°C minutes at 37°C; then 50 *μ*L of Stop solution was added to each well. Absorbance was measured at 450 nm. Sample T4 concentration was compared with the T4 standard curve and the concentration of T4 was expressed as plasma T4 concentration (in ng/mL).

### 2.4. Formulas for Calculations [45–49]:

Glucose infusion rate (GIR) is

(1) $$\begin{array}{c} GIR=\frac{glucose\;\;infusion\;\;\left(mg/min\right)}{body\;\;weight\;\;\left(kg\right)} \end{array}$$

Metabolic clearance rate of insulin (MCRI), expressed in mU/m^2^/min, is

(2) $$\begin{array}{c} MCRI=\frac{insulin\;\;infusion\;\;rate}{steady\;\;state\;\;plasma\;\;insulin\;\;concentration\;\;–\;\;basal\;\;plasma\;\;insulin\;\;concentration} \end{array}$$

Insulin sensitivity index (ISI), expressed in mg/kg/min/mU/mL, is

(3) $$\begin{array}{c} ISI=\frac{glucose\;\;infusion\;\;rate}{steady\;\;state\;\;plasma\;\;insulin\;\;concentration} \end{array}$$

Quantitative insulin sensitivity check index (QUICKI) is

(4) $$\begin{array}{c} QUICK=\frac{1}{\log\left(fasting\;\;plasma\;\;insulin\;\;\left(\mu IU/mL\right)\right)+\log\left(fasting\;\;plasma\;\;glucose\;\;\left(mg/dL\right)\right)} \end{array}$$

To estimate insulin resistance the universally accepted homeostatic model assessment (HOMA) was applied.

HOMA for insulin resistance (HOMA-IR) is

(5) $$\begin{array}{c} HOMA-IR=\frac{fasting\;\;plasma\;\;insulin\;\;\left(\mu IU/mL\right)\times fasting\;\;plasma\;\;glucose\;\;\left(mmol/L\right)}{22.5} \end{array}$$

### 2.5. Data Analysis

Statistical analysis was carried out with GraphPad Prism 7.04. All data were analyzed with one-way analysis of variance (ANOVA) followed by Tukey posttesting. In the figures, data are presented as mean ± SEM. *∗*, *∗∗*, and *∗∗∗* indicate significant difference in comparison to the healthy control group (*∗* for p<0.05, *∗∗* for p<0.01, and *∗∗∗* for p<0.001). #, ##, and ### indicate the statistically significant difference compared to the HFHSD (high-fat and high-sugar diet) control group (p<0.05, p<0.01, and p<0.001, respectively). §, §§, and §§§ indicate significant difference compared to the corresponding 0 min value (p<0.05, p<0.01, and p<0.001, respectively).

## 3. Results

**Figure 3:**
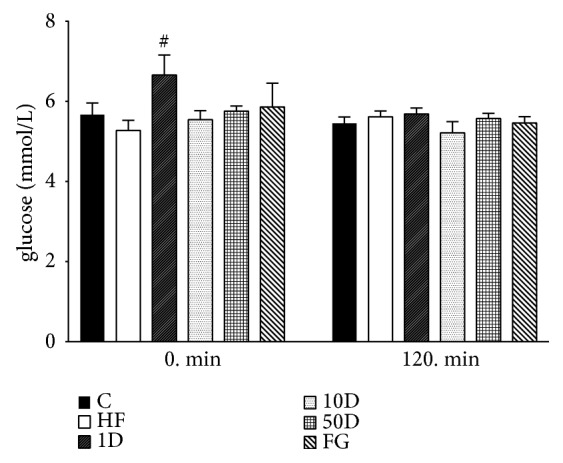
Blood glucose levels after 10 weeks of diosgenin or fenugreek treatment.

Fasting glucose levels showed statistically significant difference only between the 1D and HF group (see Figure 3). In the steady state, since in HEGC blood glucose is artificially kept in the euglycemic state, there was no significant difference between groups.

**Figure 4:**
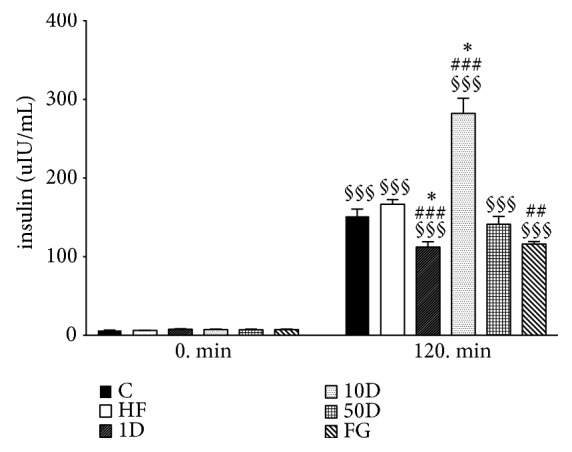
Plasma insulin levels after 10 weeks of diosgenin or fenugreek treatment.

Fasting plasma insulin levels were not statistically significant between the groups (see Figure 4). In the steady state period of HEGC due to the insulin infusion, insulin levels elevated significantly in each group compared to their respective fasting values. Furthermore, 1D animals showed a significant decrease to healthy controls and high-fat controls, while the FG group had a statistically significant decrease in insulin levels compared to the HF group only. 10D rats, however, showed a significant increase in insulin levels compared to both C and HF animals.

**Figure 5:**
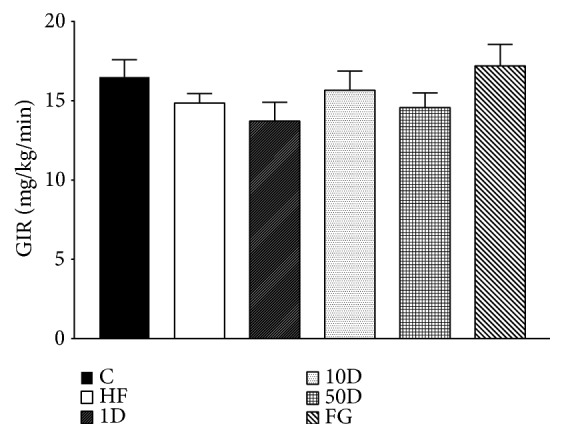
GIR after 10 weeks of diosgenin or fenugreek treatment.

There was no statistically significant difference between the groups in glucose infusion rate (see Figure 5).

**Figure 6:**
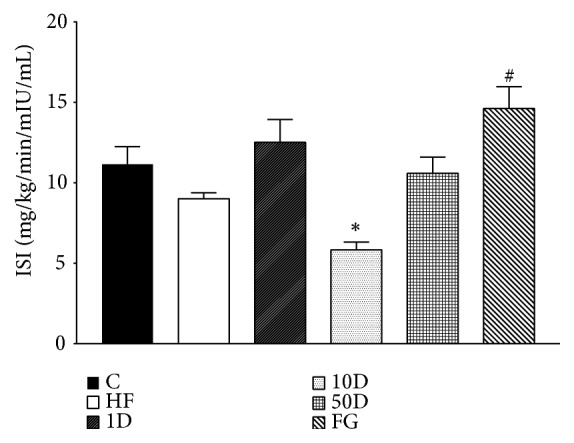
ISI after 10 weeks of diosgenin or fenugreek treatment.

In the 10D animals, insulin sensitivity index significantly decreased compared to healthy controls (see Figure 6). The FG group showed a statistically significant elevation compared to the HF rats.

**Figure 7:**
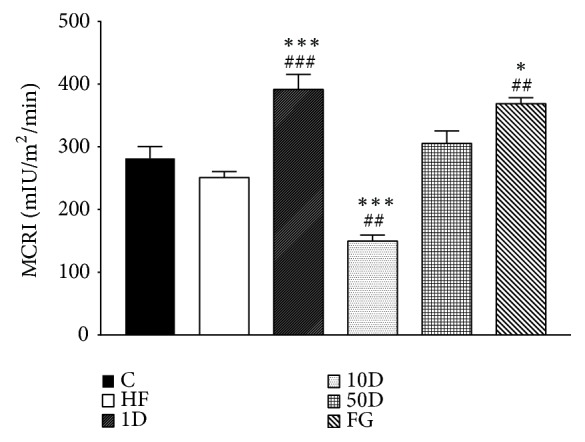
MCRI after 10 weeks of diosgenin or fenugreek treatment.

The metabolic clearance rate of insulin was significantly increased in 1D and fenugreek groups compared to both the healthy and high-fat controls (see Figure 7). 10D rats showed a statistically significant decrease in comparison to the C and HF groups.

**Figure 8:**
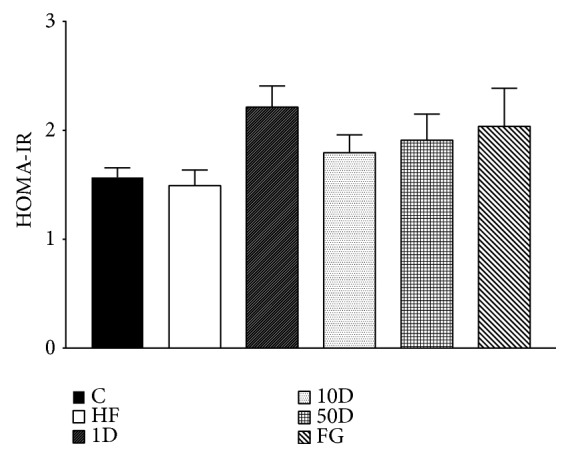
HOMA-IR after 10 weeks of diosgenin or fenugreek treatment.

In HOMA-IR, the groups showed no statistically significant difference between each other (see Figure 8).

**Figure 9:**
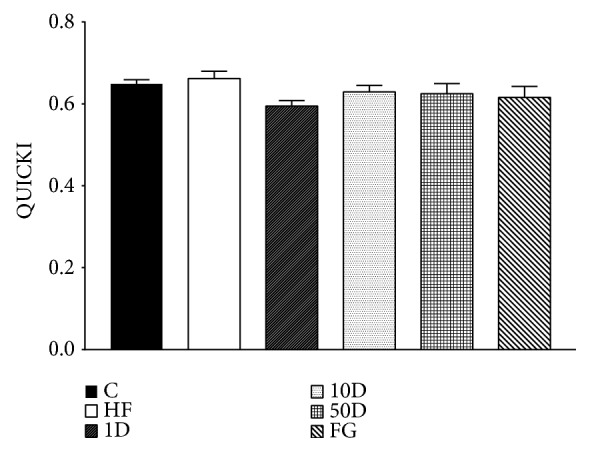
QUICKI after 10 weeks of diosgenin or fenugreek treatment.

QUICKI showed no statistically significant difference between the groups (see Figure 9).

**Figure 10:**
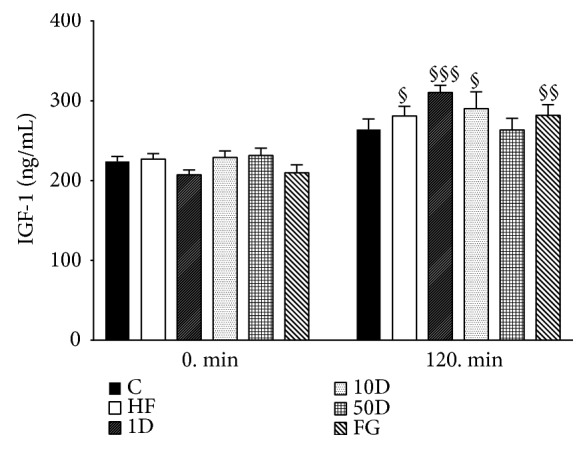
IGF-1 levels after 10 weeks of diosgenin or fenugreek treatment.

In 0 min, IGF-1 levels showed no statistically significant difference between the groups (see Figure 10). In the steady state period of HEGC, however, IGF-1 levels significantly increased in HF, 1D, 10D, and FG animals compared to their respective 0 min values.

**Figure 11:**
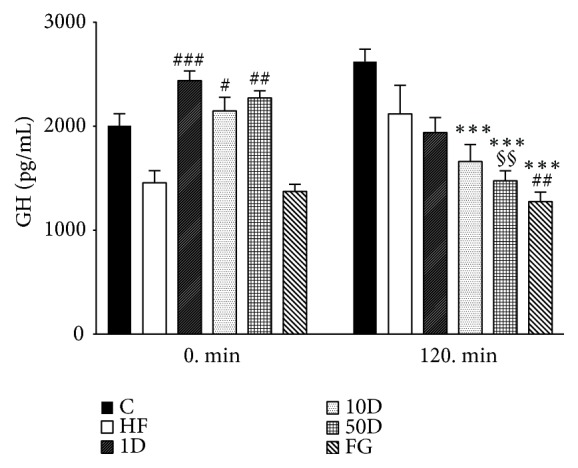
GH levels after 10 weeks of diosgenin or fenugreek treatment.

In the fasting state, all diosgenin groups, but not the FG group, showed a significant increase compared to the HF animals (see Figure 11). In the steady state period of the HEGC, however, GH levels increased in the healthy controls and the HF group, although without reaching significance compared to the corresponding 0 min value, and the diosgenin groups showed an apparent dose-dependent decrease in GH levels. With the 10D and 50D rats the decrease was statistically significant compared to healthy controls, and the 50D group also showed a significant decrease to its 0 min value. Fenugreek treatment caused a significant decrease in GH levels by the steady state period compared to both the C and HF groups.

**Figure 12:**
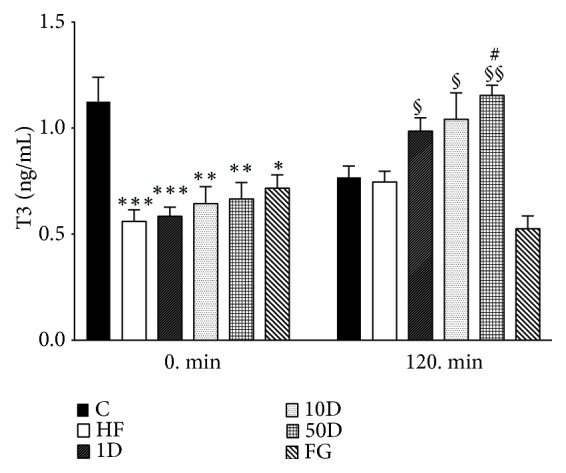
T3 levels after 10 weeks of diosgenin or fenugreek treatment.

Fasting levels of T3 were significantly decreased in all groups compared to healthy control rats (see Figure 12). During steady state, however, T3 decreased in healthy controls, despite not reaching statistical significance. The rats treated with diosgenin showed significant increase compared to their corresponding 0 min values, and the 50D group also showed a significant elevation in T3 levels compared to the steady state value of HF animals.

**Figure 13:**
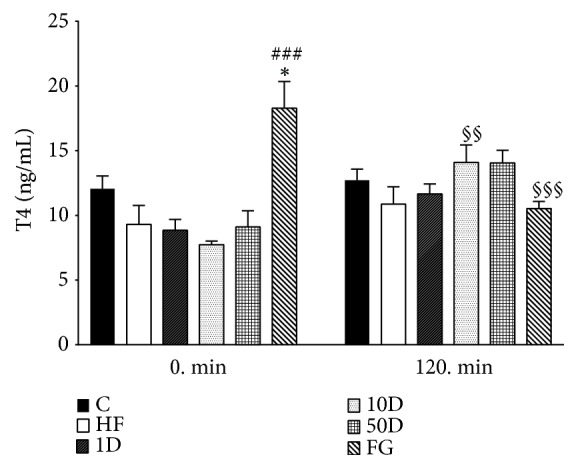
T4 levels after 10 weeks of diosgenin or fenugreek treatment.

The FG group showed a statistically significant increase in fasting T4 levels compared to healthy and high-fat controls (see Figure 13). In the steady state period of HEGC there was an apparent increase in T4 levels in the diosgenin treated animals, but only the 10D group reached statistical significance. The fenugreek seed treated rats, however, showed a significant decrease in T4 levels compared to their elevated fasting value.

## 4. Discussion

Literature data confirms that fenugreek is a widely used plant since ancient times as a spice, herb, nutritional supplement, or a therapeutic agent in different types of disorders, including diabetes mellitus, metabolic syndrome or hyperlipidaemia [3, 50–53]. It is also very effective in reducing oxidative stress damage and stimulating apoptosis in hyperplasia of different types of cells [54–58]. Both the leaves and the seeds can be administered in therapeutic or nutritional regards [53]. Some researchers and pharmaceutical manufacturers use the aqueous or alcoholic extracts of the fenugreek to enhance its effect or to prepare the best formulation of the plant. We reported in our previous study that the aqueous and alcoholic fenugreek extracts are significantly different in the profile of bioactive compounds [1]. According to our results, 4-HIL, asparagine, and various nucleotides were present only in the aqueous extract, and on the other hand the flavonoids, soyasaponins, steroidal saponins, and some vitamins such as vitamin C and B3 were detectable only in the alcoholic extract. Since the different types of extracts contain only a part of the active compounds of fenugreek; therefore, the whole plant is preferable to be administered to the healthy or diseased.

Several research data supports that besides insulin regulation fenugreek can also influence the synthesis and function of other metabolic hormones, like IGF-1, GH, T3, and T4 [59–62]. All these hormones play an important role in the regulation of glucose metabolism and the pathomechanism of diabetes mellitus. IGF-1 is by structure and function familiar to insulin, and in case of insulin deficiency it can activate the insulin receptor but the hypoglycemic effect develops at a slower rate [63]. Furthermore, IGF-1 is a very important antiapoptotic and hypertrophic agent, involved especially in the thyroid tissue hyperplasia [64–67]. Moreover, IGF-1 also stimulates the production and secretion of TSH. Both effects can contribute to the increase of thyroid hormone levels. The growth hormone is considered the most important anabolic hormone, with major role in lipolysis and the synthesis of proteins. GH plays an important role in the regulation of glucose metabolism, contributing to the development of hyperglycemia, hyperinsulinemia, and insulin resistance [68–75]. In addition, GH secretion can be modified by thyroidal hormones: in hypothyroidism GH levels and GH response is decreased [76]. Thyroidal hormones are able to influence metabolism in the whole body. They have an important role in metabolic regulation and growth, but in addition these endocrine compounds can modify cardiovascular functions, energy balance, and the central nervous system function. Thyroidal hormones greatly influence glucose metabolism either via altering IGF-1 and GH functions and increasing hepatic glucose production. Apart from being used in hypothyroidism they have indication in the treatment of depression, as well as in treatment of obesity due to the stimulation of lipolysis, but long-term usage is limited by their possible diabetes promoting and cardiovascular side effects.

During hyperinsulinemic euglycemic glucose clamp performed at the end of the six week long experimental period we found no significant differences between the groups regarding glucose infusion rate, suggesting that in response to the insulin infusion and consequent hyperinsulinemia the rate of disappearance of glucose have not changed; therefore the insulin stimulated glucose uptake was the same. However, the parameters calculated from blood samples taken in fasted and steady state suggest that the lowest dose of diosgenin and the comparable dose fenugreek seed treatment significantly improved insulin resistance. In the fenugreek group insulin sensitivity index was increased, showing that less amount of insulin was needed to achieve the same rate of disappearance of glucose from blood circulation compared to other groups. This finding is also corroborated by the increased metabolic clearance rate of insulin, suggesting that the body had to compensate against hypoglycemia by eliminating excess insulin, an effect also shown by rats treated with 1 mg/kg diosgenin. Though the latter group failed to show an increased ISI, the elevated MCRI demonstrates the improved insulin sensitivity and glucose uptake. The explanation behind the difference in insulin sensitivity effect between the fenugreek seed and the comparable dose of diosgenin might be that fenugreek might be that apart from diosgenin; fenugreek seeds contain other active compounds that can influence glucose metabolism. One such compound is the newly identified 4-hydroxyisoleucine (4-HIL). Several studies demonstrated that 4-HIL is able to enhance insulin sensitivity via different molecular targets, such as AMP-activated protein kinase, suppressor of cytokine signalling-3, insulin receptor substrate-1, and in some reports showing an effect comparable to that of metformin [77–79]. The 50 mg/kg diosgenin treatment failed to alter insulin sensitivity, but rats treated with 10 mg/kg diosgenin showed a marked drop in ISI and MCRI, suggesting insulin resistance in peripheral tissues. This seemingly contradictory response compared to the lowest dose of diosgenin might be explained with the phenomena of hormesis, a relatively new, but not well understood theory in toxicology that states that certain compounds in low dose have a stimulating, but in high doses, an opposite, inhibitory effect. Hormesis might be explained if we assume that in low doses the toxic compound activates adaptive responses in the body, thus achieving a beneficial effect, but in higher doses these adaptive mechanisms cannot counterbalance the stress and undesired effects manifest [80–82]. Based on our results we suggest that, in 1 mg/kg dose, diosgenin has an insulin sensitizing effect, but in 10 mg/kg dose it promotes insulin resistance.

Continuous insulin infusion effectively increased IGF-1 levels in the high-fat control, 1 and 10 mg/kg diosgenin, as well as in the fenugreek groups. IGF-1 elevation might have appeared as a result of hyperinsulinemia and the significantly increased GH levels. In response to elevated basal GH levels IGF-1 increased by the steady state period, activating growth hormone inhibiting hormone (GHIH), and, in turn, decreasing GH levels that can contribute to normalization of blood glucose levels [83]. Hyperinsulinemia also stimulates thyroid hormone production and secretion, and IGF-1 levels elevate in the thyroid gland, possibly as a result of elevated GH [84]. IGF-1 stimulates TSH and the proliferation of thyroid tissue that leads to increased secretion of thyroid hormones and consequent hypoglycemia. In the state of high plasma glucose levels less T4 prohormone is transformed into T3, T3/T4 ratio decreases, and obesity might develop [60]. Our results showed that in chronic diosgenin and fenugreek treatment the basal level of thyroid hormones, especially T3 significantly decreased, but the consequent IGF-1 elevation in response to hyperinsulinemia in the steady state effectively balances this reduction in the diosgenin groups. The FG rats showed a significant elevation of basal T4 levels, but in the steady state period the insulin infusion reduces T4, possibly through stimulating its transformation into T3 due to elevated GH that promotes hypothyroidism [85, 86]. Our results corroborate that there are complex feedback mechanisms and interplay between the studied anabolic hormones that play an important role in the regulation of glucose metabolism, development of insulin resistance, and modulating insulin sensitivity. Our additional find is that fenugreek seed may have different physiological effects than its active component diosgenin due to the fact that apart from compounds with potentially therapeutic value it might also contain molecules that cause undesired or even toxic side effects, especially in long-term treatment. However, it is important to note the significance of proper dosing, since low dose had beneficial effects, but elevation of doses impaired insulin sensitivity and the homeostasis of the examined hormones.

## 5. Conclusion

Our study shed light to that chronic consumption of fenugreek seed is able to influence the complex interplay of anabolic hormones. Our results also indicate that apart from its proven insulin sensitizing effect fenugreek might have a therapeutic potential in the adjuvant treatment of thyroid diseases. We plan to conclude further research to determine the effect of fenugreek and its saponin agents in different thyroid disease models.

## Data Availability

The authors confirm that all relevant data are presented in the results section of this publication. Nonetheless, the raw data that support the findings of this work are available from the corresponding author upon reasonable request.
